# Development of a bispecific CDH17-GUCY2C ADC bearing the ferroptosis inducer RSL3 for the treatment of colorectal cancer

**DOI:** 10.1038/s41420-025-02652-0

**Published:** 2025-07-28

**Authors:** Ying Zhang, Jing Du, Xiaohong Cui, Yuhang Ling, Chengwu Tang

**Affiliations:** 1 Zhejiang University School of Medicine, Hangzhou, 310058 China; 2 Central Laboratory, First Affiliated Hospital of Huzhou University, Huzhou, 313000 China; 3 Huzhou Key Laboratory of Translational Medicine, First People’s Hospital of Huzhou, Huzhou, 313000 China; 4 Key Laboratory of Digital Technology in Medical Diagnostics of Zhejiang Province, First People’s Hospital of Huzhou, Huzhou, 313000 China; 5 Key Laboratory of Aerospace Medicine of Ministry of Education, School of Aerospace Medicine, Fourth Military Medical University, Xi’an, 710032 China; 6 Psychiatry Department, Shanxi Bethune Hospital, Taiyuan, 030032 China; 7 Department of Hepatopancreatobiliary Surgery, First Affiliated Hospital of Huzhou University, Huzhou, 313000 China

**Keywords:** Cell death, Cancer therapy

## Abstract

Colorectal cancer is a malignant tumor of the colon or rectum, with approximately 150,000 new cases each year. Current treatment strategies, such as surgery, chemotherapy, radiotherapy, and immunotherapy, face challenges ranging from cancer recurrence, drug resistance to significant toxicity. Therefore, these patients urgently need more effective treatments. Ferroptosis, a novel form of cell death characterized by iron-dependent lipid peroxidation, has emerged as a promising new approach for treating colorectal cancer. Inactivation of phospholipid hydroperoxide glutathione peroxidase (GPX4) or the cysteine/glutamate antiporter SLC7A11 leads to the depletion of cellular glutathione (GSH), resulting in lipid peroxidation and subsequent ferroptosis. Here, we found that CDH17 and GUCY2C are co-overexpressed in colorectal cancer cells and developed a bispecific antibody-drug conjugate (BsADC) targeting CDH17 and GUCY2C, conjugated with the ferroptosis inducer RSL3 (a GPX4 inhibitor). Experimental results showed that, compared to monoclonal antibody ADCs, BsADC exhibits better binding and internalization activities, and could inhibit tumor cell proliferation more effectively. Moreover, the BsADC displayed an advantageous safety profile in mice. These findings suggest that the CDH17-GUCY2C BsADC, which induces ferroptosis in tumor cells, could be a promising new treatment for colorectal cancer.

## Introduction

Colorectal cancer (colorectal cancer) is the third most common cancer globally and the second leading cause of cancer death [1, 2]. Current treatments for colorectal cancer mainly include laparoscopic surgery for primary disease, more aggressive excision for metastatic disease, radiation therapy for rectal cancer, and neoadjuvant and palliative chemotherapy [3–5]. However, the impact of these new treatment options on cure rates and long-term survival has been limited. Although monoclonal antibodies such as cetuximab, panitumumab, bevacizumab, ramucirumab, and trastuzumab in combination with single or multi-agent chemotherapeutics have displayed clinical benefits in advanced colorectal cancer and significantly improved the overall survival of patients up to 30 months [6–10]. Patients’ overall survival rate is still low compared to other cancers. Antibody-drug conjugates (ADCs) are one of the fastest-growing biological therapeutics in the field of oncology, showing promising therapeutic effects in many types of cancer [11]. However, their clinical application in colorectal cancer has shown limited success [12, 13]. Therefore, discovering new drug targets and developing novel therapeutics, especially ADCs, are extremely important for effectively treating such cancers.

CDH17 is a cell adhesion molecule that plays an essential role in maintaining epithelial cell polarity and tissue homeostasis, primarily by mediating calcium-dependent cell adhesion [14]. However, CDH17 is overexpressed in various adenocarcinomas, including colorectal, gastric, and pancreatic cancers [15–17]. High levels of CDH17 are associated with metastatic disease and poor prognosis in patients with these malignancies, which has sparked interest in this protein as a diagnostic and therapeutic target [18]. In normal cells, CDH17 is only expressed in the apical membrane. Still, due to the loss of cell polarity in tumor cells, CDH17 is distributed across the surface of tumor cells, which provides a good opportunity for targeted therapy [19]. Several bispecific antibodies, antibody-drug conjugates (ADCs), and CAR-T products targeting CDH17 are under clinical investigation [16, 20].

Glucoside cyclase C (GUCY2C) is a type I transmembrane receptor that regulates intestinal homeostasis, tumorigenesis, and obesity when activated by its hormone ligand [21]. GUCY2C is expressed in the epithelial cells of the normal intestine and is enriched on the apical side of these cells [21]. Similar to CDH17, it is significantly overexpressed in colorectal cancer and is distributed across the apical membrane and basolateral membrane of tumor cells, disrupting the tight junctions of the tumor cells, which is beneficial for interaction with targeted therapies [22]. The expression pattern of GUCY2C makes it a suitable antigen for colorectal tumors. Previous studies have reported that a bispecific antibody targeting GUCY2C and a CAR-T cell therapy showed strong anti-tumor efficacy, demonstrating the feasibility of developing GUCY2C-targeted therapies [23].

Ferroptosis is a unique form of cell death that depends on the Fe^2+^, reactive oxygen species (ROS), and phospholipids with polyunsaturated fatty acid chains (PUFA-PLs) [24]. Recently, it has attracted considerable attention due to its unique differences in morphology and mechanisms compared to other types of programmed cell death. An increasing number of studies in recent years have shown that inducing ferroptosis in colorectal cancer cells by increasing intracellular Fe^2+^ and ROS levels, decreasing the levels of the antioxidant glutathione (GSH), or inactivating GPX4 can contribute to the clinical treatment of colorectal cancer, while inhibiting ferroptosis may lead to tumor progression and therapeutic resistance in colorectal cancer [25, 26]. Therefore, modulating ferroptosis may be a promising strategy for the treatment of colorectal cancer. Additionally, cancer cells resistant to conventional therapies are particularly susceptible to ferroptosis [27]. Many studies indicate that modulating ferroptosis pathways can enhance the anti-tumor effects of colorectal cancer therapeutics [28, 29]. Similarly, both in vitro and in vivo experiments show that using pharmacological agents Ras-selected lethal 3 (RSL3) that trigger ferroptosis-induced cancer cell death holds promise in colorectal cancer [30, 31].

Here, we generated a bispecific antibody targeting CDH17 and GUCY2C, and conjugated it with the ferroptosis inducer RSL3 to create an innovative ADC molecule. The CDH17-GUCY2C BsADC demonstrated promising anti-tumor activity alongside favorable toxicological profiles. Our findings suggest that the CDH17-GUCY2C BsADC could be an innovative therapeutic agent in colorectal cancer treatment.

## Results

### CDH17 and GUCY2C are co-overexpressed in colorectal tumors

**Fig. 1:**
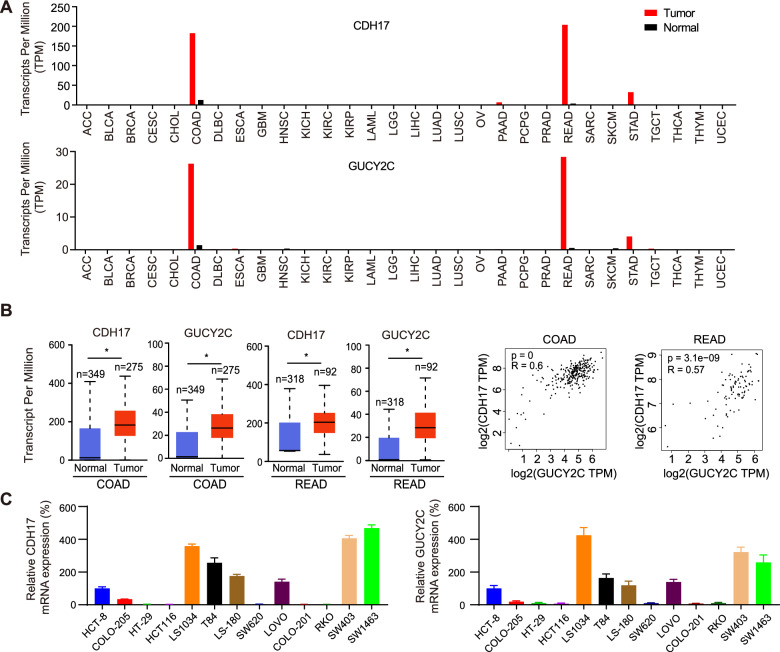
CDH17 and GUCY2C are co-expressed at high levels in colorectal cancer. **A** Expression of RNA transcription of CDH17 and GUCY2C in the GEPIA cancer tissue sample database. **B** CDH17 and GUCY2C are co-expressed at high levels in colon adenocarcinoma (COAD) and rectum adenocarcinoma (READ). Tumor samples are shown in red, normal samples are shown in gray, and the corresponding sample numbers are indicated. The correlation analysis results also indicate that CDH17 and GUCY2C are highly expressed concurrently in the tumors of the majority of patients. **C** Relative RNA expression of CDH17 and GUCY2C in different tumor cell lines. RNA from 13 tumor cells was isolated, and the expression of CDH17 and GUCY2C was detected by RT-PCR.

A comprehensive analysis was conducted on the Gene Expression Profiling and Interactive Analyses (GEPIA) database to identify antigens as potentially suitable tumor-associated targets for developing BsADCs with CDH17, which should also be highly expressed in colorectal cancer. In the spectrum of 33 human cancer types, GUCY2C demonstrates a similar expression pattern to CDH17 (Fig. 1A). We also found that CDH17 and GUCY2C were co-high expressed in most colorectal cancer patients and rectum adenocarcinoma patients. We further analyzed the expression of CDH17 and GUCY2C in the GEPIA database. As shown in Fig. 1B, both CDH17 and GUCY2C are highly expressed in colorectal cancer. We also detected the expression of CDH17 and GUCY2C in 13 tumor cell lines by RT-PCR, and found that CDH17 and GUCY2C have a pronounced co-expression in various tumor cells (Fig. 1C). These results indicate that there is a strong correlation between the expression levels of CDH17 and GUCY2C in colorectal tumor cells, providing support for the construction of BsADCs targeting CDH17 and GUCY2C.

### Generation and characterization of CDH17 X GUCY2C BsAbs

**Fig. 2:**
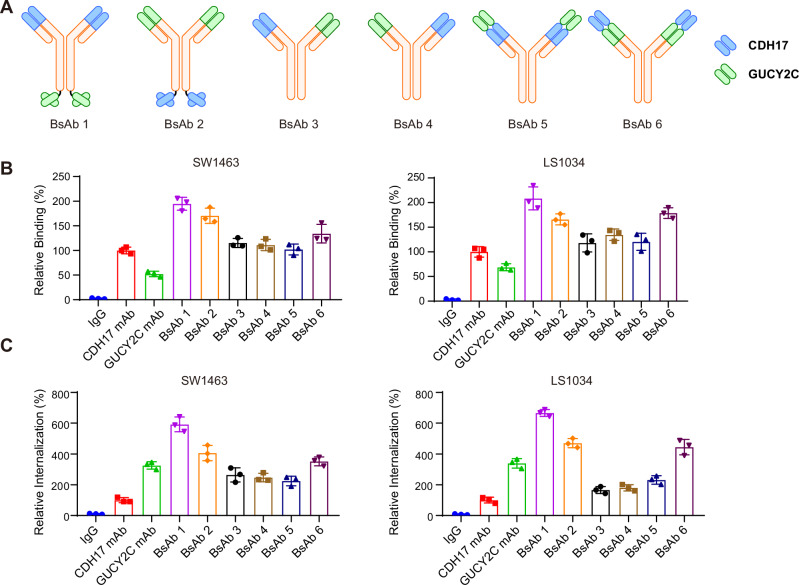
Development and characterization of CDH17 x GUCY2C BsAbs. **A** Structure diagram of six different CDH17 x GUCY2C BsAb formats. Knob-into-hole and crossmab technologies are used respectively to prevent mismatching of heavy and light chains in format BsAb 3 and BsAb 4. **B** The binding activities of CDH17 x GUCY2C BsAbs were analyzed in SW1463 and LS1034 cell lines at a concentration of 1 μg/mL. The CDH17 and GUCY2C monoclonal antibodies were used as controls. **C** The internalization activities of CDH17 x GUCY2C BsAbs were analyzed in SW1463 and LS1034 cell lines at a concentration of 1 μg/mL. The CDH17 and GUCY2C monoclonal antibodies were used as controls.

We constructed the BsAbs using the CDH17 and GUCY2C antibody sequences from two clinical products, BI-905711 and PF-07062119. To obtain a BsAb with high binding and internalization activities, we constructed six bispecific antibodies with different structures (Fig. 2A). BsAb1 and BsAb2 use a “2 + 2” quadrivalent structure, in which the C-terminal of one monoclonal antibody is linked to the SCFv form of the other antibody. BsAb3 and BsAb4 use a “1 + 1” bivalent structure, and we used the “Knob-into-hole” and the “CrossMab” techniques to avoid the mismatch of heavy and light chains. BsAb5 and BsAb6 also adopt a tetravalent structure, with the difference being that the variable region sequences of both antibodies are located at the N-terminus.

Next, we examined the binding and internalization activities of these six CDH17 x GUCY2C bsAbs in SW1463 and LS1034 cancer cells. As shown in Fig. 2B, BsAb1 demonstrated superior binding activities in both cells compared to the parent monoclonal antibodies. At the same time, we also found that the CDH17 monoclonal antibody had better binding activity than the GUCY2C monoclonal antibody, which was probably caused by the higher expression level of CDH17 on tumor cells than GUCY2C. However, the endocytic activity of the CDH17 monoclonal antibody is much lower than that of the GUCY2C monoclonal antibody, which may reduce the anti-tumor activity of the CDH17 monoclonal antibody ADC. But when combined with GUCY2C to form BsAbs, several bispecific structures showed improved endocytic activity, with BsAa1 exhibiting the best endocytic activity among them (Fig. 2C). These data suggested that BsAb1 with a “2 + 2” quadrivalent structure could enhance the binding and internalization activities of tumor cells, which was suitable for the construction of BsADC.

### CDH17 X GUCY2C BsADC exhibits significant anti-tumor activity in vitro

**Fig. 3:**
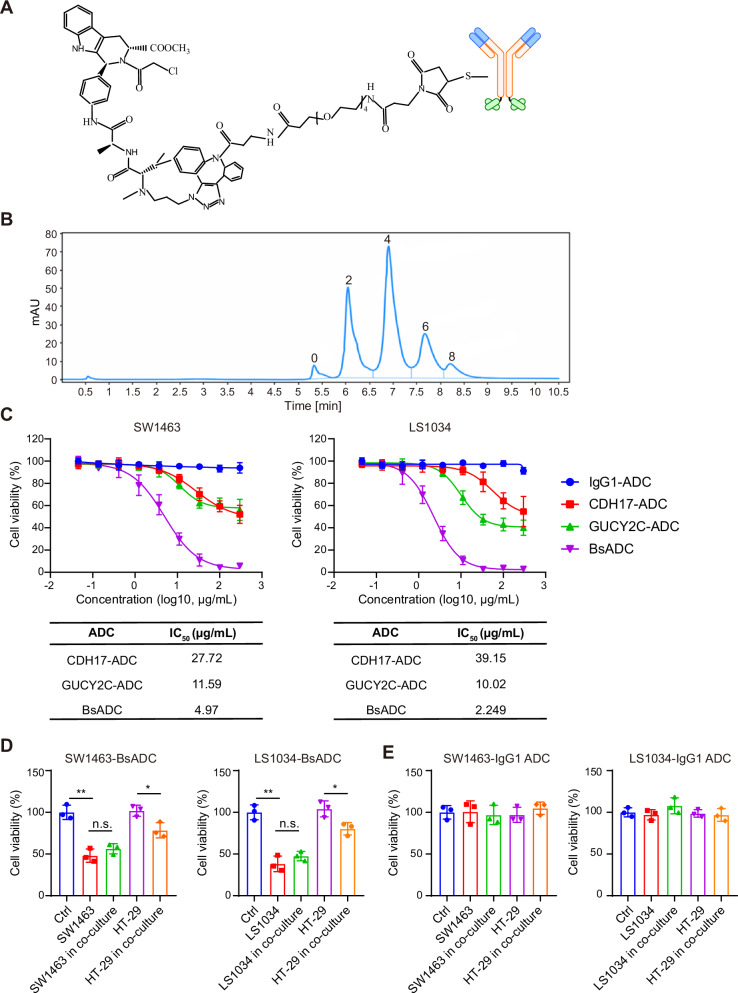
CDH17 x GUCY2C BsADCs demonstrate potent anti-tumor efficacy in vitro. **A** The chemical structure of CDH17 x GUCY2C BsADC, composed of BsAb, an Ala-Val linker, and the ferroptosis inducer payload RSL3-NH2. The linker and payload were conjugated to BsAb with an average drug/antibody ratio of 4. **B** The conjugated drug distribution by the reverse phase chromatographic analysis. **C** In vitro cytotoxicity of CDH17 x GUCY2C BsADCs and control ADC against SW1463 and LS1034 cells. The cancer cells were treated with ADC molecules for 72 h, and cell viability was measured with a CCK-8 kit. Bystander killing assay. Cytotoxicity induced on CDH17/GUCY2C positive cells (SW1463, LS1034), CDH17/GUCY2C negative cells HT-29 and SW1463/HT-29 or LS1034/HT-29 co-cultures with 5 µg/mL of CDH17 x GUCY2C BsADC (**D**) or IgG1-ADC (**E**). *, *p* < 0.05; **, *p* < 0.01.

RSL3 was identified as an activator of ferroptosis by inhibiting glutathione peroxidase 4 (GPX4). The CDH17 x GUCY2C BsADCs were generated by bio-conjugating the RSL3-NH2 payload to endogenous cysteine residues on BsAbs following a previously published paper [32]. Hydrophobic interaction chromatography showed a homogeneous drug distribution, and the DAR of synthesized ADC was approximately four (Fig. 3B). At the DAR of 4, the molar concentration of RSL3 conjugated with 1 μg CDH17-ADC or GUCY2C-ADC was about 26.67 nM, while the molar concentration of RSL3 conjugated with 1 μg BsADC was about 20 nM.

We next examined the cytotoxic effects of BsADCs in SW1463 and LS1034 cells, which highly express both CDH17 and GUCY2C. We observed that the inhibitory activity of BsADCs on tumor cell proliferation was dose-dependent, and this activity was significantly higher than that of the monospecific ADCs targeting CDH17 and GUCY2C individually (IC_50_, 4.97 μg/mL vs 27.72 μg/mL and 11.59 μg/mL in SW1463, 2.249 μg/mL vs 39.15 μg/mL and 10.02 μg/mL in LS1034) (Fig. 3C). In contrast, the non-binding control ADC (IgG-ADC) containing the identical linker and payload did not exhibit cell growth inhibitory activities (Fig. 3C). These results indicate that the BsADC can specifically inhibit tumor cells with high expression of CDH17 and GUCY2C.

As the standard chemotherapy regimen for the treatment of CRC, 5-fluorouracil (5-FU) is listed as a first-line basic medication in international guidelines. To evaluate the therapeutic potential of the ferroptosis inducer RSL3, we compared the cytotoxic effects of RSL3 and 5-FU. The results show that the IC_50_ of RSL3 (2.342 μM in SW1463, 1.494 μM in LS1034) is significantly lower than that of 5-FU (164 μM in SW1463, 7.309 μM in LS1034), which further demonstrates the potential of using RSL3 as a payload (Supplementary Fig. 1).

A distinctive advantage of antibody-drug conjugates (ADCs) compared to conventional immune-oncology therapies lies in their capacity to induce bystander killing effects [33, 34]. To test whether CDH17 × GUCY2C BsADC exhibited bystander cytotoxicity, we evaluated the ability of CDH17 x GUCY2C BsADC to induce bystander cytotoxicity to CDH17/GUCY2C negative cells (HT-29) after mixing with CDH17/GUCY2C positive cells (LS1034, SW1463) for 72 h. As shown in Fig. 3D, when SW1463 or LS1034 cells were co-cultured with HT-29, the killing amount of SW1463 or LS1034 cells did not increase, but compared with the control group, co-culture with SW1463/HT-29 or LS1034/HT-29 increased the bystander kill of CDH17/GUCY2C negative HT-29 cells. In contrast, SW1463/HT-29 or LS1034/ HT-29 incubated IgG1-ADC for 72 h showed no significant change in cytotoxicity (Fig. 3E).

### CDH17 X GUCY2C BsADC induces ferroptosis in colorectal tumor cells

**Fig. 4:**
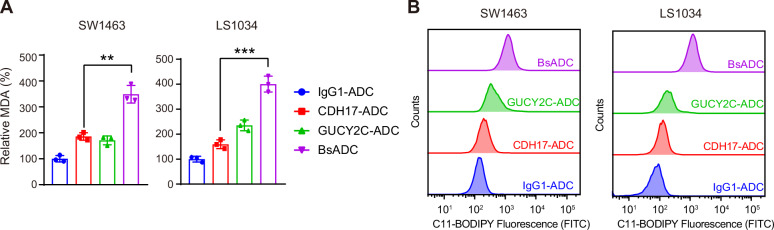
CDH17 x GUCY2C BsADC induced ferroptosis in colorectal cancer cells. **A** BsADC induced the MDA accumulation in SW1463 and LS1034 cells. **B** BsADC promoted the lipid ROS accumulation in SW1463 and LS1034 cells, which was analyzed by flow cytometry with BODIPY-C11 staining. **, *p* < 0.01; ***, *p* < 0.001.

We then examined whether BsADC induced cell death by ferroptosis. SW1463 and LS1034 cells were treated with 5 µg/mL of hIgG1-ADC, CDH17-ADC, GUCY2C-ADC, and BsADC for 24 h, followed by the assessment of relevant ferroptosis markers. Ferroptosis is often accompanied by Malondialdehyde (MDA) accumulation within cells. Therefore, we examined MDA level in our cell samples. We found that cells treated with BsADC exhibited a markedly higher level of both MDA than those treated with hIgG1-ADC, CDH17-ADC, and GUCY2C-ADC (Fig. 4A). Additionally, using BODIPY-C11 staining to visualize ROS changes in our cell samples allowed us to further investigate alterations in lipid peroxidation levels. As shown in Fig. 4B, the lipid peroxidation products in tumor cells were significantly increased after BsADC treatment and were significantly more than those in cells treated with CDH17-ADC or GUCY2C-ADC. Taken together, these results indicate that BsADC induced ferroptosis by transporting RSL3 into tumor cells.

### CDH17 X GUCY2C BsADC has good anti-tumor activity and safety profile in mice model

**Fig. 5:**
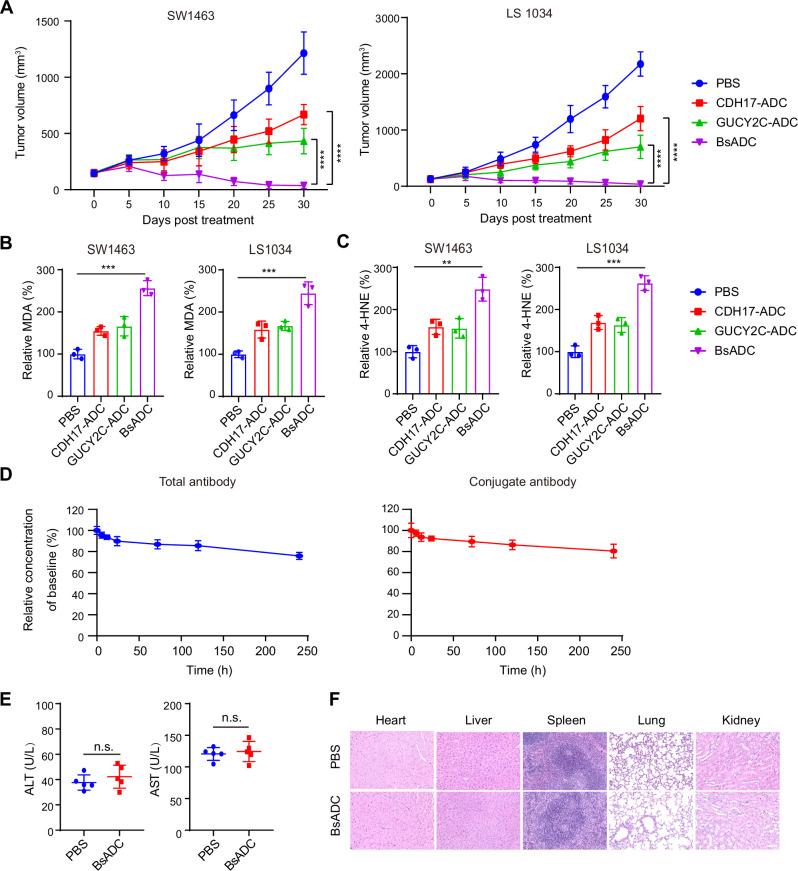
CDH17 x GUCY2C BsADC shows promising efficacy and safety in mouse models. **A** The anti-tumor activity of CDH17 x GUCY2C BsADC in SW1463 and LS1034 xenograft models. Corresponding cancer cells were injected subcutaneously into 7-week-old female immunodeficient mice (5 mice per group) at 5 × 10^6^ cells per mouse, and CDH17-ADC (1 mg/kg), GUCY2C-ADC (1 mg/kg), and BsADC (1 mg/kg) were intravenously administered when the tumor volume of the mice reached a volume of approximately 100–200 mm^3^. Tumor volumes were measured. The relative levels of MDA (**B**) and 4-HNE (**C**) in tumor tissues were measured. **D** Stability of CDH17 x GUCY2C BsADC in human serum. The serum stability of CDH17 x GUCY2C BsADC was evaluated in PHS over a period of 10 days. The concentrations of both total antibody and conjugated antibody were monitored at various time points. **E** 7-week-old female hCDH17/hGUCY2 double-humanized BALB/c mice were intravenously injected with PBS or CDH17 × GUCY2C BsADC (1 mg/kg), once every 5 days for a total of 7 injections. The blood chemistry parameters (ALT, left, AST, right) 1 day after the last injection. Data are presented as mean values ± SD. **F** Representative images of H&E stained the heart, liver, spleen, lung, and kidney tissues of the mice (Scale bar: 100 μm). **, *p* < 0.01; ***, *p* < 0.001; ****, *p* < 0.0001.

We further evaluated the in vivo anti-tumor activity of CDH17 x GUCY2C BsADC in a mouse xenograft tumor model. Mice transplanted with SW1463 and LS1034 cancer cell lines were injected intravenously with CDH17-ADC, GUCY2C-ADC, or CDH17 x GUCY2C BsADCs. A comparison of tumor growth showed that CDH17 x GUCY2C BsADC treatment resulted in significant tumor regression compared to CDH17-ADC or GUCY2C-ADC treatment (Fig. 5A). Moreover, we observed that CDH17 x GUCY2C BsADC treatment resulted in increased MDA and 4-HNE levels in tumor tissue compared to CDH17-ADC or GUCY2C-ADC treatment (Fig. 5B, C). This result is consistent with the results of in vitro cell growth inhibition experiments, and also indicates that the BsADC can deliver more RSL3 into the tumor cells to induce ferroptosis.

In order to determine the stability of CDH17 x GUCY2C BsADC in human serum in vitro, total antibody and conjugated antibody concentrations were measured at different times. After incubation for 10 days, the concentration of total antibodies in human serum decreased to the initial amount of 75.84% ± 3.04%, while the concentration of conjugation antibodies would decrease to the initial amount of 80.44% ± 6.32% (Fig. 5D).

We further characterized the nonclinical safety profile of the CDH17 x GUCY2C BsADC in mice. To assess potential toxicity, we quantified the levels of aspartate aminotransferase (AST) and alanine aminotransferase (ALT), which are indicators of liver function, and found that these enzymes remained within the normal range (Fig. 5E). These results suggest that CDH17 x GUCY2C BsADC does not induce hepatotoxicity. Similarly, no signs of target-dependent toxicity were observed in multiple normal tissues examined, including the heart, liver, spleen, lungs, and kidneys (Fig. 5F). These findings demonstrate that the CDH17 x GUCY2C BsADC exhibits both potent anti-tumor effects and favorable safety profiles in mouse models, reinforcing its potential as a treatment option for colorectal cancer.

## Discussion

The current treatment methods for CRC include surgical intervention, chemotherapy, radiotherapy, targeted drug therapy, and immunotherapy. Still, the therapeutic effects of these methods are not satisfactory, and patients often end up with tumor recurrence. The 5-year survival rate for patients with advanced CRC is significantly below 14% [35, 36]. Cancer cells have an increased demand for metabolic intermediates required for energy production to maintain their rapid proliferation, producing reactive oxygen species (ROS). At the same time, cancer cells also respond to high levels of ROS by enhancing their antioxidant defense mechanisms to resist cell death [37]. Regulators that induce ferroptosis can directly or indirectly affect GPX4 activity by modulating various metabolic pathways, decreasing cellular antioxidant capacity, and accumulating lipid reactive oxygen species (ROS), ultimately leading to ferroptotic cell death [38]. Currently, some studies have used combinations of natural products or FDA-approved drugs to induce ferroptosis in colorectal cancer (CRC). For example, a KRAS (G12C) inhibitor approved by the FDA in 2021 can induce ferroptosis in colorectal cancer cells [39]. Similarly, cetuximab, which targets the epidermal growth factor receptor (EGFR), enhances the effect of RSL3 in inducing ferroptosis in KRAS-mutant CRC cells by activating p38 MAPK and inhibiting the NRF2/HO-1 axis [40]. Furthermore, the combined use of cetuximab and β-elemene, a bioactive compound isolated from the Chinese herb Curcumae Rhizoma, sensitizes KRAS-mutant CRC cells by inducing ferroptosis, accompanied by ROS accumulation and GSH depletion [41]. Overall, these studies suggest that promoting ferroptosis in tumor cells is an effective means of treating colorectal cancer.

Although many small molecule ferroptosis inducers have been proven to effectively inhibit the growth of colorectal cancer, these small molecules often lead to toxic side effects due to the lack of targeting [42]. ADCs are designed to conjugate cytotoxic payloads to antibodies, which can specifically bind to tumor surface antigens and be internalized, releasing the toxins inside tumor cells to exert their cytotoxic effects. Therefore, ADCs can directly target and deliver cytotoxic drugs to tumors, thereby minimizing toxicity to normal tissues [43, 44]. Existing ADCs for colorectal cancer mainly use microtubule inhibitors (such as auristatins and maytansine) and DNA-damaging agents (such as calicheamicin and duocarmycin) as toxins [45]. GPX4 and SLC7A11 are the primary targets for inducing ferroptosis in CRC [26]. In addition to RSL3 inhibiting CRC by suppressing GPX4 and generating ROS, several other small molecules can promote ferroptosis in colorectal cancer cells. For example, 2-imino-6-methoxy-2H-chromene-3-carbothioamide (IMCA) reduces the expression of SLC7A11 and depletes cysteine and GSH, which decreases the activity of colorectal cancer cells in vitro and inhibits tumor growth in vivo [46]. Acyl-CoA Dehydrogenase Short/Branched Chain (ACADSB) negatively regulates the expression of GPX4 and enhances the accumulation of Fe^2+^ and lipid peroxidation in colorectal cancer cells, thereby inducing ferroptosis [47]. Apatinib reduces the expression of GPX4 and upregulates the expression of ACSL4 and ECOVL6 to promote ferroptosis in colorectal cancer cells [48]. In the future, more ferroptosis inducing small molecules can be used to treat colorectal cancer in the form of ADCs.

Currently, ADCs targeting colorectal cancer are mainly monoclonal antibody ADCs, with primary targets including human epidermal growth receptor 2 (HER2), guanylyl cyclase C (GCC), mesothelin, and carcinogenic antigen-related cell adhesion molecule 5 (CEACAM5), and so on [49–52]. Additionally, some new colorectal cancer-specific targets have been identified, and related ADCs have shown significant anti-tumor effects in preclinical experiments, such as the seven-transmembrane receptor protein GPR56 [53]. However, there is limited research on BsADCs for colorectal cancer. Compared to monoclonal ADCs, BsADCs can target two tumor-specific antigens simultaneously, significantly increasing the binding activity of the antibody to tumor cells [54]. The CDH17 x GUCY2C BsADC also shows better binding activity than CDH17 or GUCY2C monoclonal ADCs. Moreover, the endocytic activity of the CDH17 monoclonal antibody is weak; although it is highly expressed in tumors, ineffective internalization leads to weak anti-tumor activity. The GUCY2C monoclonal antibody has intense endocytic activity, which allows the CDH17 x GUCY2C BsADC to have better endocytic activity, thereby achieving better anti-tumor activity.

The structural formats of BsAb directly affects the number and distribution of its antigen-binding sites and influences the drug efficacy. The asymmetric 1 + 1 antibody format is similar to the natural antibody and has the lowest immunogenicity [55]. However, antibodies exist in the monovalent formats of two antigens, and their binding and endocytosis are weaker than those in the 2 + 2 formats. In addition, the connection mode of antigen-binding sites in the 2 + 2 formats is also one of the key factors affecting the efficacy of drugs. By linking scFv to the N-terminus of conventional antibodies, the molecule binds four antigens simultaneously, increasing the potency of a single antibody, and the two binding sites are too close to each other, which increases steric hindrance and may simultaneously destroy the optimal binding of both targets and reduce the functional potency [56, 57]. In clinical, scFv that form up-down structures by fusion at the C-terminus of traditional antibodies are commonly used and have good affinity and efficacy, such as cadonilimab (AK104) against PD1 x CTLA-4 BsAb and BL-B01D1 (EGFR x HER3-ADC) against 2 + 2 up-down structures [58, 59]. Fortunately, both CDH17 and GUCY2C are expressed at low levels in normal tissues, and BsADC do not increase binding and internalization to normal tissues, thus avoiding toxicity. At present, BsADCs have already entered the clinical phase and have achieved good therapeutic effects, such as the use of EGFR-HER3 BsADCs for the treatment of lung cancer [60]. In addition, several EGFR x cMET BsADCs targeting various solid tumors are also under clinical investigation [61–63]. It is hoped that in the future, BsADCs will also bring new treatment methods to more patients with colorectal cancer.

## Materials and methods

### Cell lines and culture conditions

The human HEK293T (ATCC®CRL-3216), COLO-205 (ATCC®CCL-222), HCT-8 (ATCC®CCL-244), HT-29 (ATCC^®^HTB-38), HCT116 (ATCC®CCL-247), T84 (ATCC®CCL-248), LS1034 (ATCC®CRL-2158), LS-180 (ATCC®CL-187), SW620 (ATCC^®^CCL-227), LOVO (ATCC®CCL-229), COLO-201 (ATCC®CCL-224), RKO (ATCC®CRL-2577), SW1463 (ATCC®CCL-234), SW403 (ATCC®CCL-230) cell lines were obtained from the American Type Culture Collection (ATCC) (Manassas, VA, USA). All cells were cultured in DMEM or RPMI-1640 Medium containing 10% fetal bovine serum (FBS; Invitrogen), 1% penicillin-streptomycin (Gibco-BRL), and 2 mM L-glutamine at 37 °C with 5% CO_2_. Following the manufacturer’s instructions, Transfections were performed using the Lipofectamine 3000 transfection kit (Thermo Fisher) according to the manufacturer’s instructions. All cells were confirmed to be free of mycoplasma contamination by using a cell culture contamination detection kit (Thermo). None of the cell lines used in this study was found in the database of commonly misidentified cell lines maintained by ICLAC and NCBI Biosample.

### Generation of CDH17 X GUCY2C BsAbs

We selected the CDH17 antibody sequence of BI-905711 and the GUCY2C antibody sequence of PF-07062119 to develop the BsAbs. The BsAbs were generated in-house using the Knob-into-hole and the CrossMab techniques to avoid the mismatch of heavy and light chains [64]. The constructs were transfected into the HEK293E cells according to the published transient transfection procedure [65]. Two weeks posttransfection, Recombinant proteins were concentrated from clarified cell culture supernatants through a process of centrifugation and filtration. Subsequently, antibody constructs were purified from the supernatant using protein A chromatography, following established protocols [66]. The antibodies were analyzed by SDS-PAGE and SEC to ensure that their purity was better than 95%.

### Generation and characterization analysis of CDH17 X GUCY2C BsADC

CDH17 x GUCY2C-RSL3-NH2 antibody-drug conjugate was performed as previously described [32]. Briefly, under the conditions with EDTA, the CDH17 x GUCY2C bispecific antibody was treated with TCEP to decrease the interchain disulfide bonds. Maleimide-PEG4-DBCO in DMSO was then added to the reduced antibody solution to form the CDH17 x GUCY2C-DBCO complex. Finally, the CDH17 x GUCY2C-DBCO complex was mixed with N3-Val-Ala-RSL3-NH2 and incubated at 25 °C to yield the CDH17 x GUCY2C-RSL3-NH2 antibody-drug conjugate. N3-Val-Ala-RSL3-NH2 was synthesized as described previously [32].

### RNA extraction, cDNA synthesis, and real-time PCR analysis

Total RNA was extracted from cultured cells using the RNeasy Mini Kit (Qiagen 74104), and 1 µg of RNA was reverse-transcribed into cDNA with a kit from Promega, following the provided protocols. Quantitative real-time PCR was performed with iQ SYBR Green Master Mix (Bio-Rad) on the CFX96 Touch Real-Time PCR Detection System, with samples processed and analyzed accordingly. The gene expression levels were normalized to actin. The primer sequences used for PCR were: CDH17 forward, 5′-TCTTACCCGAGAGGGATCTC-3′ and CDH17 reverse, 5′-CCTTCCTGGTCAATTGAAAATGG-3′; GUCY2C forward, 5′-CTGAAGGGTGACCGAGCA-3′ and GUCY2C reverse, 5′-CCAGTGGATTCCATCCTAGAGG-3′; actin forward, 5′-GCTCGTCGTCGACAACGGCT-3′ and actin reverse, 5′-CAAACATGATCTGGCTCATCTTCTC-3′.

### Cytotoxic assay in vitro

Cell viability was evaluated using the CCK-8 kit (MCE, HY-K0301). The SW1463 and LS1034 cell lines were plated into 96-well plates and incubated overnight at 37 °C. Following a 72 h treatment with the ADCs, the CCK-8 kit was employed to measure the cytotoxicity of the ADCs by determining cell viability according to the manufacturer’s instructions. Absorbance readings at 450 nm were measured using a microplate reader.

### Cell surface binding measured by FACS

To assess the antibody’s binding affinity to tumor cells, the bispecific antibody (1 μg/mL) was incubated with the tumor cells, and the fluorescence intensity of the antibody was quantified using a BD flow cytometer. The acquired data were subsequently analyzed with Flow-JO software to determine the antibody’s binding capacity.

### Internalization assay

pHAb reactive dyes (Promega, G9841) were utilized to track receptor-mediated antibody endocytosis. Following the manufacturer’s instructions, antibodies were conjugated with the pHAb dyes. Diluted to a concentration of 1 μg/mL, these conjugated antibodies were incubated with tumor cells. During receptor-mediated endocytosis, the antibody-pHAb complexes are transported to the acidic environment of endosomes and lysosomes, triggering the fluorescence of the pHAb dyes. Flow cytometry was employed to measure this fluorescence.

### Malondialdehyde (MDA) assay

MDA concentrations in cell or tissue lysates were measured using the MDA Assay Kit (Dojindo, M496). Samples (3 × 10^6 cells or 30 mg tissue in 200 μL lysis buffer) were reacted with 250 μL test solution at 95 °C for 15 min. The absorbance reading of the supernatant was measured at 532 nm.

### Lipid peroxidation (4-HNE) assay

Lipid Peroxidation (4-HNE) Assay Kit (Abcam, ab238538) were used to evaluate the concentration of 4-HNE according to the manufacturer’s protocol. Briefly, samples were first applied to plates coated with 4-HNE, followed by detection antibody and HRP-labeled secondary antibody, and absorbance was detected at 450 nm by a chromogenic reaction.

### Lipid ROS measurement

Lipid ROS level was examined by BODIPY™ 581/591 C11 (Thermo, #D3861). Briefly, at the end of treatment, cells were incubated with BODIPY at 1 μM final concentration for 20 min at 37 °C in the dark. After three times of PBS washing, lipid ROS levels were analyzed using flow cytometry.

### Bystander killing assay

To investigate the effects of ADCs on cell viability, CDH17/GUCY2C-positive cells (LS1034, SW1463) and CDH17/GUCY2C-negative cells (HT-29) stably transfected with pCDH-CMV-MCSEF1-copGFP plasmid were mixed at a 1:1 ratio (40,000 cells per well) in 6-well plates. After overnight incubation, the cells were treated with 5 µg/mL of CDH17 x GUCY2C BsADC or IgG1-ADC. Following a 72 h incubation, the cells were harvested, centrifuged, and stained with propidium iodide (2 µL of a 500 µg/mL stock in PBS) to identify dead cells. Cell viability was assessed by flow cytometry, with the percentage of live cells reported as the mean ± SEM relative to untreated cells (set as 100% viability). At least three independent experiments were conducted.

### Growth inhibition of tumor xenografts in vivo

NU/NU Nude mice were acquired from Vital River Laboratories (Beijing). All animal studies followed institutional guidelines of the Animal Care and Use Committee (IACUC) of the Medical Research and Clinical Trial Ethics Committee of Huzhou First People’s Hospital. The SW1463 and LS1034 tumor cells (5 × 10^6^ cells) were implanted subcutaneously into female immunodeficient mice (7 weeks old). Once tumor size reached a volume of approximately 100–200 mm^3^, the tumor-bearing mice were randomized to achieve equal average tumor size and variance in the treatment group (*n* = 5). ADCs were injected intravenously twice at 5-day intervals for a total of 7 injections. Tumor volume was measured each time with calipers (1/2 x length x width^2^).

### In vitro serum stability

The serum stability of CDH17 x GUCY2C BsADC in vitro was evaluated by adding 10 µg/mL CDH17 x GUCY2C BsADC to sterilized human serum filtered by a 0.22 µM filter and treated with penicillin-streptomycin (100 µL, 100 units). Incubation at 37 °C. Serum samples were collected at 0 h, 6 h, 12 h, 1 d, 3 d, 5 d and 10 d, respectively. Assess the drug concentration, including total antibody and conjugated antibodies.

### Toxicological analysis in vivo

The hCDH17/hGUCY2 double-humanized BALB/c mice were acquired from Biocytogen (Beijing). 7-week-old female hCDH17/hGUCY2 double-humanized BALB/c mice (*n* = 5) were administered either vehicle control (PBS) or BsADC at a dosage of 1 mg/kg, once every five days for a total of 7 injections. One day after the last injection, serum was collected from mice to detect alanine aminotransferase (ALT) and aspartate aminotransferase (AST) levels. Mice were subsequently sacrificed, the heart, liver, spleen, lung, and kidney were isolated intact, gently rinsed 2–3 times with pre-chilled PBS to remove residual tissue, and rapidly transferred to 4% paraformaldehyde for fixation. After paraffin embedding, sections were prepared and stained with H&E staining to assess the potential non-specific toxicity of BsADC to major organs of mice.

### Statistical analysis

Statistical analyses were conducted using GraphPad Prism software (version 8). Each cell line experiment was independently repeated more than three times, with each condition tested in technical triplicate. Data are presented as means ± SD. Differences between groups were assessed using ANOVA with Tukey’s post hoc test. A *p* value of less than 0.05 was considered statistically significant.

## Supplementary information

Supplementary information

## Data Availability

The datasets used and analyzed during the current study are available from the corresponding author upon reasonable request.
