# Supplementary material for: Development of a bispecific CDH17-GUCY2C ADC bearing the ferroptosis inducer RSL3 for the treatment of colorectal cancer
Source: Cell Death Discov. 2025 Jul 28;11:347. doi: 10.1038/s41420-025-02652-0 (PMC12304242; doi:10.1038/s41420-025-02652-0)
Supplement: Supplementary file 1 — Supplementary information [file 41420_2025_2652_MOESM1_ESM.docx]

Supplemental Figures

Development of a bispecific CDH17-GUCY2C ADC bearing the ferroptosis inducer RSL3 for the treatment of colorectal cancer


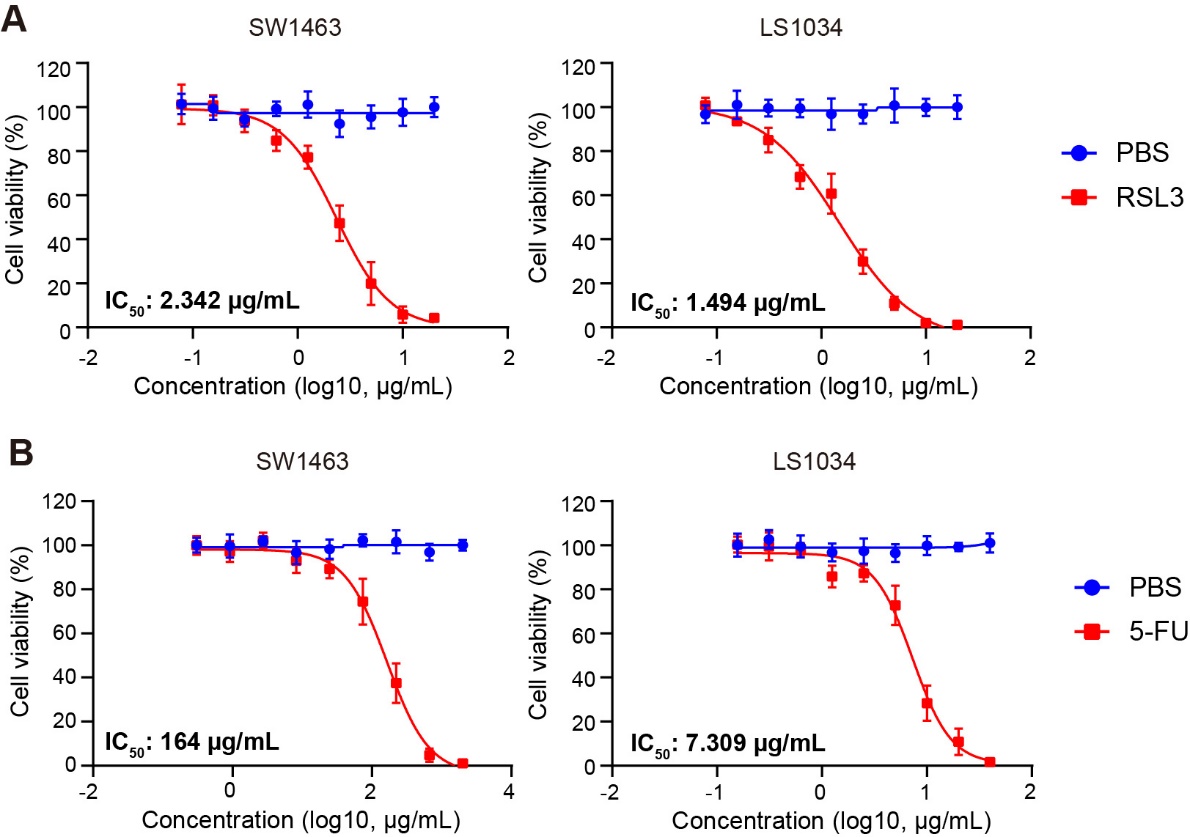


**Supplementary Fig. 1 RSL3 induces superior** **cytotoxicity compared to 5-FU.**

*In vitro* cytotoxicity of RSL3 (A) and 5-FU (B) against SW1463 and LS1034 cells. The cancer cells were treated with RSL3 or 5-FU for 72 h, and cell viability was measured with a CCK-8 kit.
